# Ouabain at nanomolar concentrations is cytotoxic for biliary tract cancer cells

**DOI:** 10.1371/journal.pone.0287769

**Published:** 2023-06-30

**Authors:** Christian Mayr, Tobias Kiesslich, Dino Bekric, Marlena Beyreis, Michael Kittl, Celina Ablinger, Elen Neureiter, Martin Pichler, Felix Prinz, Markus Ritter, Daniel Neureiter, Martin Jakab, Heidemarie Dobias

**Affiliations:** 1 Center of Physiology, Pathophysiology and Biophysics, Institute of Physiology and Pathophysiology Salzburg, Paracelsus Medical University, Salzburg, Austria; 2 Department of Internal Medicine I, University Clinics Salzburg, Paracelsus Medical University, Salzburg, Austria; 3 Institute of Pharmacy, Paracelsus Medical University, Salzburg, Austria; 4 Division of Oncology, Department of Internal Medicine, Medical University of Graz, Graz, Austria; 5 Research Unit for Non-Coding RNA and Genome Editing, Medical University of Graz, Graz, Austria; 6 Translational Oncology, University Hospital of Augsburg, Augsburg, Germany; 7 Ludwig Boltzmann Institute for Arthritis und Rehabilitation, Paracelsus Medical University, Salzburg, Austria; 8 Gastein Research Institute, Paracelsus Medical University, Salzburg, Austria; 9 Kathmandu University School of Medical Sciences, Dhulikhel, Nepal; 10 Institute of Pathology, University Clinics Salzburg, Paracelsus Medical University, Salzburg, Austria; 11 Cancer Cluster Salzburg, Salzburg, Austria; Universidade Federal do Rio de Janeiro, BRAZIL

## Abstract

Biliary tract cancer is a deadly disease with limited therapeutic options. Ouabain is a well-known inhibitor of the pumping function of Na^+^/K^+^-ATPase, though there is evidence that low concentrations of ouabain lead to a reduction of cell viability of cancer cells independent of its inhibition of the pumping function of the Na^+^/K^+^-ATPase. Regarding the impact of ouabain on biliary tract cancer, no data is currently available. Therefore, we aimed for a first-time investigation of ouabain as a potential anti-neoplastic biliary tract cancer agent using comprehensive human biliary tract cancer in vitro models. We found that ouabain has a strong cell line-dependent cytotoxic effect with IC_50_ levels in the (low) nanomolar-range and that this effect was not associated with the mRNA expression levels of the Na^+^/K^+^-ATPase *α*, *β* and *fxyd*-subunits. Regarding the mode of cytotoxicity, we observed induction of apoptosis in biliary tract cancer cells upon treatment with ouabain. Interestingly, cytotoxic effects of ouabain at sub-saturating (< μM) levels were independent of cellular membrane depolarization and changes in intracellular sodium levels. Furthermore, using a 3D cell culture model, we found that ouabain disturbs spheroid growth and reduces the viability of biliary tract cancer cells within the tumor spheroids. In summary, our data suggest that ouabain possesses anti-biliary tract cancer potential at low μM-concentration in 2D and 3D in vitro biliary tract cancer models and encourage further detailed investigation.

## 1 Introduction

Biliary tract cancer (BTC) is an epithelial disease arising from different locations within the biliary tree [1]. Based on the anatomic location of the tumor, BTCs are classified into intrahepatic cholangiocarcinoma, perihilar cholangiocarcinoma, extrahepatic cholangiocarcinoma and gallbladder cancer [2, 3]. Although BTC is relatively rare, it represents the second most common primary liver cancer after hepatocellular carcinoma. On a molecular level, BTCs harbor a complex landscape of genetic aberrations which affects, among others, cell cycle regulation, signal transduction and apoptosis response [2]. Moreover, it is well established that several epigenetic mechanisms are deregulated in BTC, including DNA methylation, histone modifications and expression of non-coding RNAs [2, 4–8]. As symptoms are rather unspecific, most patients are diagnosed at an already advanced stage of the disease, hence, therapeutic options are limited and the five-year survival rate remains poor [1]. Moreover, despite all efforts, the incidence and mortality for specific BTC subtypes is increasing globally [2, 9]. Currently, surgery is the only treatment option with curative intent, however, for most patients, only palliative treatment is possible, which mainly comprises a combination of the chemotherapeutics cisplatin and gemcitabine as first-line and FOLFOX as second-line option [10, 11]. Therefore, investigation of new therapeutic strategies remains an important part of current research activities regarding BTC.

The Na^+^/K^+^-ATPase (NKA) is an enzyme that is essential for animal cell function by maintaining sodium and potassium homeostasis across the plasma membrane. The NKA is crucial in generation and maintenance of the resting membrane potential as well as in establishing and maintaining ion gradients for secondary active transport processes [12, 13]. The NKA consists of the two core subunits *α* and *β* as well as potential accessory *fxyd* proteins, where the *α* subunit represents the catalytic (ion translocating) part of the NKA and the *β* subunit has regulatory function regarding Na^+^ and K^+^ binding affinities as well as sorting of the pump to the cell membrane [12]. Up to now, a total number of four α subunits (*α1–4*), three β subunits (*β1–3*) and seven *fxyd* isoforms were identified [14, 15]. Usually, NKA consists of a heterodimer of an *α* and a *β* subunit. The *fxyd* isoforms are not essential for the pumping function of the NKA, but are believed to be important regulators of NKA function, and they are expressed in a tissue-specific manner [12].

Ouabain is a cardiotonic steroid which, at saturating concentrations (μM- to mM range), inhibits NKA pumping activity (‘ionic pathway’) and thereby increasing heart muscle contractility [13]. However, it has been shown that nanomolar concentrations of ouabain (so-called sub-saturating concentrations) exert effects on the NKA independent of the inhibition of its pumping function, but rather by activation of the NKA as a signal transducer (‘signaling pathway’) [16]. Haas et al. revealed that treatment of myocytes with ouabain resulted in activation of Src kinase as well as increased interaction between *Src* and *EGFR* with subsequent phosphorylation of *EGFR* and activation the *Ras*/*MAPK* cascade [17, 18]. Moreover, Liang et al. suggest that two different pools of NKAs exist, one that possesses ion-pumping function and one that possesses signal transduction / receptor function and that NKAs of both pools are susceptible towards ouabain binding [19]. Interestingly, the signal transduction function of NKA seems dependent on the *α1* subunit, as knockdown of this subunit mainly resulted in reduction of the signal transduction NKA pool and not the ion pumping NKA pool [19]. Further indication for the existence of a separate signal transduction NKA pool was reported by Wang et al., who have shown that the ouabain-induced NKA-*Src* complexes are assembled at specific invaginations of the plasma membrane termed caveolae, suggesting that this NKA pool serves for signal transduction [20]. Interestingly, the effect of ouabain-induced NKA signaling appears to be dependent on the malignancy status of the cell [16]. Li et al. demonstrated that ouabain at nanomolar-concentrations protects kidney cells from apoptosis and stimulates their proliferation [21]. Likewise, ouabain protected heart muscle cells from cardiotoxic effects at low doses as well as rat cortical neurons from apoptosis induction [22, 23]. However, nanomolar concentrations of ouabain were shown to have cytotoxic and anti-proliferative effects in cancer cells. Kometiani et al. observed that treatment of breast cancer cells with 100 nM ouabain inhibited proliferation independent of inhibition of the pumping function of the NKA [24]. Moreover, this effect was accompanied by stimulation of the *Src*/*EGFR* interaction and thus activation of NKA signaling [24]. In human glioblastoma cells, ouabain caused a hybrid cell death of apoptosis and necrosis [25]. Similar results regarding a general cytotoxic effect of ouabain have been described for several other cancer entities [12, 26–29]. However, up to now, the effects of ouabain on BTC cells are unknown. Therefore, the aim of this study is a first-time investigation of the potential of ouabain as an anti-BTC substance.

## 2 Materials and methods

### 2.1. Cell culture and substances

BTC cell lines EGi-1 (ACC 385, RRID:CVCL_1193, [30]) and TFK-1 (ACC 344, RRID:CVCL_2214, [31]) were purchased from the German Collection of Microorganisms and Cell Cultures (DSZM; Braunschweig, Germany). BTC cell lines HuCCT-1 (JCRB0425, RRID:CVCL_0324, [32]), KKU-055 (JCRB1551, RRID:CVCL_M258), KKU-100 (JCRB1568, RRID:CVCL_3996, [33]), NOZ (JCRB1033, RRID:CVCL_3079, [34]), OCUG-1 (JCRB0191, RRID:CVCL_3083, [35]) and OZ (JCRB1032, RRID:CVCL_3118, [36]) were purchased from the Japanese Collection of Research Bioresources Cell Bank (JCRB, Osaka, Japan). The non-tumor cholangiocyte cell line MMNK-1 was purchased from JCRB (JCRB1554, RRID:CVCL_M266). Cells were cultured in a cell incubator (37° C, humidified atmosphere, 5% CO_2_) using high-glucose Dulbecco’s modified Eagle’s medium (DMEM, Gibco, ThermoFisher Scientific, Waltham, MA, USA) supplemented with 10% (v/v) fetal bovine serum (FBS, Biochrom, Berlin, Germany), 1% antibiotic–antimycotic (ABAM, Merck, Darmstadt, Germany), 1 mM sodium pyruvate (Pan Biotech, Aidenbach, Germany), and 10 mM HEPES (Pan Biotech). Ouabain was purchased from Merck (Darmstadt, Germany) as powder, dissolved in water into a stock concentration of 10 mM and stored in aliquots at -20°C.

### 2.2. Cell viability

For measurement of the cytotoxicity of ouabain, cells were seeded in transparent 96-well microplates using a seeding number of 10,000 cells per well and let grown for 24 hours. Cells were then washed with serum-free DMEM and incubated with a 10-step 1:2 dilution series of ouabain starting at 5 μM (5 μM– 0.01 μM) for 72 hours in serum-free DMEM. Viability was measured using the resazurin assay as described [37] and a Spark multimode reader (Tecan, Grödig, Austria). IC_50_-values were calculated by four-parameter logistic regression.

For time-resolved analysis of the cytotoxicity of ouabain, HuCCT-1, OCUG-1 and TFK-1 cells were seeded in transparent 96-well microplates using a seeding number of 10,000 cells per well and let grown for 24 hours. Cells were washed with serum-free DMEM and incubated with a 10-step 1:2 dilution series of ouabain starting at 1 μM (1 μM– 0.002 μM). Viability was measured after 0, 24, 48 and 72 hours, respectively, using the resazurin assay and a Spark multimode reader. Visual evaluation of cells was performed using an inverted, phase-contrast microscopy (CKX53) and documented using a XM10 camera (Olympus Europe, Hamburg, Germany).

### 2.3. Caspase 3/7 activity assay

For the Caspase-Glo®3/7 Assay (Promega, Mannheim, Germany), HuCCT-1, OCUG-1 and TFK-1 cells were seeded in transparent 96-well microplates using a seeding number of 10,000 cells per well and let grown for 24 hours. Cells were washed with serum-free DMEM and incubated with 1 μM, 0.1 μM and 0.01 μM ouabain, respectively, as well as with 10 μM staurosporine (Selleckchem, Houston, TX, USA) as a positive control for 24 hours. Caspase 3/7 activity was measured according to the manufacturer’s instructions on a Spark multimode reader.

### 2.4. RealTime-Glo™ Annexin V Apoptosis and Necrosis Assay

For the RealTime-Glo™ Annexin V Apoptosis and Necrosis Assay, HuCCT-1, OCUG-1 and TFK-1 cells were seeded in a white-walled clear bottom 96-well microplate using a seeding number of 10,000 cells per well and let grown for 24 hours. Cells were washed with serum-containing DMEM and incubated with 1 μM, 0.1 μM and 0.01 μM ouabain in serum-containing DMEM. Detection reagent was added in serum-containing DMEM according to the manufacturer’s instructions. The plate was placed in a Spark multimode reader for 50 hours using environmental control (37°C, 5% CO_2_) to simulate cell incubator conditions. To avoid evaporation, the plate was placed in a humidity cassette and empty wells and spaces on the plate were filled with PBS. Luminescence and fluorescence were measured every 30 minutes.

### 2.5. Annexin-V/7-AAD staining

For Annexin-V/7-AAD staining, cells were seeded in 35-mm petri dishes at a density of 4×10^5^ cells per dish and grown for one day. Following a washing step with serum-free DMEM, cells were treated with 0.01, 0.1, or 1.0 μM ouabain or left untreated. 4, 24 or 48 hours post-treatment, the cell culture supernatants were transferred into 15 ml tubes and cells were harvested using Trypsin/EDTA. The cell suspensions were centrifuged for five minutes at 232 g, supernatants were discarded, and the cell pellets were resuspended in 100 μl Annexin-V binding buffer (BioLegend, Inc., San Diego, CA, USA) plus 5 μl FITC (fluorescein isothiocyanate)-conjugated Annexin-V and 5 μl 7-AAD (7-aminoactinomycin D) staining solution (BioLegend, Inc.). After 15 minutes of staining at room temperature in the dark, binding buffer was added to each sample to a final volume of 400 μl and cells were used immediately for flow cytometry in a BD Accuri C6 Plus flow cytometer equipped with a CSampler Plus auto-sampler (BD Biosciences). Fluorescence emissions of FITC-Annexin-V on FL1 (533 nm band pass filter) and 7-AAD on FL3 (670 nm long pass filter) were measured upon excitation with a 488 nm argon laser. Unstained and single-stained samples were used for establishing FL1–FL3 compensation settings. 80,000–180,000 cells were analyzed in each sample using FlowJo software version 10.6.2 (BD Biosciences) and depicted in FL3 versus FL1 dot plots. Spider gates were set to segregate cells in four different populations: Annexin-V^−^/7-AAD^−^cells were considered as non-apoptotic, Annexin-V^+^/7-AAD^−^cells as early apoptotic, Annexin-V^+^/7-AAD^+^ cells as late apoptotic, and Annexin-V^–^/7-AAD^+^ cells as post-late apoptotic/necrotic.

### 2.6. Measurement of Lactate Dehydrogenase (LDH) release

Induction of necrosis was evaluated using an LDH-Glo ™Cytotoxicity Assay (Promega). HuCCT-1, OCUG-1 and TFK-1 cells were seeded in a transparent 96-well microplate using a seeding number of 10,000 cells per well and let grown for 24 hours. Cells were washed with serum-free DMEM and incubated with 1 μM, 0.1 μM and 0.01 μM ouabain, respectively for 24 hours. 15 minutes prior addition of the LDH detection reagent, 10% Triton X-100 (Merck) was added as a positive control. LDH-levels were measured via luminescence according to the manufacturer’s protocol using a Spark multimode reader.

### 2.7. Cellular membrane potential assay

For the Cellular Membrane Potential Kit (abcam, Cambridge, United Kingdom), HuCCT-1, OCUG-1 and TFK-1 cells were seeded in a black-walled clear-bottom 96-well microplate using a seeding number of 10,000 cells per well and let grown for 24 hours. Cells were washed with serum-free DMEM and incubated with the dye-loading solution for 45 minutes at room temperature protected from light according to the manufacturer’s protocol. Then, cells were incubated with 1 μM, 0.1 μM and 0.01 μM ouabain as well as 1 mM ouabain and 100 mM K^+^ as controls for induction of membrane depolarization and 1 μM valinomycin as a control for membrane hyperpolarization. Immediately upon incubation, the plate was placed in a Spark multimode reader and fluorescence was measured every two minutes for 90 minutes (excitation 605 nm / emission 650 nm according to the manufacturer’s instructions using environmental control (37°C, 5% CO_2_) to simulate cell incubator conditions and a humidity cassette to avoid evaporation.

### 2.8. Patch clamp

For patch clamp experiments, HuCCT-1, OCUG-1 and TFK-1 cells were seeded on 0.01% poly-D-lysine (PDL) -coated coverslips (Ø 12 mm) and cultured for at least 24 h in DMEM. Coverslips were transferred to a RC-25 recording chamber (Warner Instruments, Hamden, CT, USA) and mounted on a Nikon Eclipse TE2000-U inverted microscope. Experiments were performed at room temperature in the whole-cell perforated patch clamp mode using 130 μM amphotericin to the pipette solution. Recordings were started as soon as the serial resistance was below 30 MΩ for the perforated configuration. Patch electrode resistances were 4–9 MΩ. After establishing the whole-cell configuration, cells were superfused with an extracellular solution and data were recorded using an EPC-10 amplifier controlled by PatchMaster software (HEKA, Lambrecht/Pfalz, Germany). Cell membrane potential (V_mem_) recordings were performed in the zero-current clamp mode. The intracellular (pipette) solution contained (in mM): 70 K_2_SO_4_, 10 NaCl, 10 KCl, 4 MgCl_2_, 2 CaCl_2_, 5 HEPES free acid (FA), 10 EGTA (249 mOsm/kg, pH 7.2 adjusted with KOH). The extracellular solution contained (in mM): 140 NaCl, 5.6 KCl, 2.5 CaCl_2_, 1.5 MgCl_2_, 10 HEPES FA, 4.5 glucose and mannitol, which was used to achieve isotonic conditions between the extracellular solution and the media of cells (300–340 mOsm/kg, pH 7.4 adjusted with NaOH). Ouabain (1 μM, 0.3 μM, 0.1 μM, 0.01 μM) was added to the extracellular solution as indicated in the individual experiments. Bath solution exchange was performed with a valve-controlled gravity-driven perfusion system (ALA Scientific Instruments, Farmingdale, NY, USA) at a flow rate of 3–5 ml/min. Osmolalities of intra- and extracellular solutions were measured using a vapor pressure osmometer (Wescor, Logan, UT, USA). Ouabain at 500 μM or 1 mM was used as positive control to induce cell membrane depolarization.

### 2.9. Intracellular sodium assay

HuCCT-1, OCUG-1 and TFK-1 cells were seeded in a 96-well black microplate with transparent bottom at 10,000 cells per well and let grown over night. For determination of intracellular Na^+^ levels, the Brilliant Sodium Flex kit (ION Biosciences, San Marcos, TX, USA) was used according to the manufacturer’s instructions. After one hour of staining in dye loading buffer containing Probenecid, the loading buffer was removed and 50 μl of wash buffer were added to each well. Experimental conditions were established by adding 50 μl of an extracellular solution composed of (in mM) 140 NaCl, 5.6 KCl, 2.5 CaCl_2_, 1.5 MgCl_2_, 10 HEPES FA, 4.5 glucose, 1 mM sodium pyruvate and 5.0 mannitol (305 mOsm/kg, pH 7.4 adjusted with NaOH) containing 2× concentrations of ouabain to obtain final concentrations of 1 mM, 1 μM, 0.1 μM and 0.01 μM. Untreated cells and cells exposed to the monovalent cation ionophore Gramicidin (5 μM final concentration from a 1 mM stock in ethanol) in a high (150 mM) extracellular K^+^ solution and 1 mM ouabain served as controls. Immediately after induction, a kinetic measurement was started using a Tecan Spark multimode plate reader at 37°C. Fluorescence bottom readings were made every 90 seconds for 180 minutes (excitation 485 nm / emission 545 nm). A baseline fluorescence bottom measurement prior to establishing experimental conditions was used as a reference readout.

### 2.10. Mitochondrial membrane potential

Mitochondrial membrane potential was measured using the JC-1 dye (Merck). HuCCT-1, OCUG-1 and TFK-1 cells were seeded in transparent 96-well microplates using a seeding number of 10,000 cells per well and let grown for 24 hours. Cells were washed with serum-free DMEM and incubated with 1 μM, 0.1 μM and 0.01 μM ouabain for 6 hours. Prior to staining, JC-1 was warmed in a water bath, vortexed and centrifuged at 17,000 g for 10 minutes as described [38]. After incubation, cells were washed with serum-free media and 5 μM staining solution in Hanks’ Balanced Salt Solution (HBSS; ThermoFisher Scientific, Waltham, MA, USA) were added. The positive control Valinomycin (Tocris, Bristol, United Kingdom) was added with the JC-1 dye to avoid prolonged incubation and cytotoxicity. For staining, cells were incubated for 30 minutes in a cell incubator. Then, cells were washed twice with HBSS and fluorescence was measured at excitation 485 nm / emission 525 nm (green) and excitation 480 nm / emission 590 nm (red) using a Spark multimode reader. Changes of mitochondrial potential were calculated as ratio of red to green fluorescence.

### 2.11. NKA subunit expression analysis

Expression analysis of NKA subunits was done via measurement of mRNA levels. In short, cells were seeded in 60 mm dishes and let grown for 24 hours. Cells were washed with PBS and harvested using Tri Reagent® (Merck). RNA was isolated via a Direct-zol™ RNA MiniPrep (Zymo Research, Irvine, CA, USA) according to the manufacturer’s instructions. cDNA was generated using a GoScript™ Reverse Transcriptase Kit (Promega). Quantitative real-time PCR was performed on a ViiA7 real-time PCR system (Applied Biosystems, ThermoFisherScientific) using GoTaq® Master Mix (SYBR® Green, Promega) using the following protocol: initial denaturation (95°C, 2 minutes) followed by 45 cycles (95°C for 3 seconds and 60°C for 30 seconds). Each biological replicate contained four technical replicates. A melting curve analysis was performed to guarantee the specificity of the products. mRNA expression levels of NKA subunits are presented as ΔCt values related to beta-actin. Primers were purchased as 100 μM stocks (KiCqStart® SYBR® Green Primers, Merck). Primer sequences are listed in S1 Fig.

### 2.12. Autophagy assay

For autophagy assay, cells were seeded in a black 96-well microplate with transparent bottom using a seeding number of 15,000 cells per well (HuCCT-1, OCUG-1) or 10,000 cells per well (TFK-1) and let grown for 24 hours. Cells were then washed with serum-free DMEM and incubated with ouabain (1 μM, 0.1 μM or 0.01 μM) or rapamycin (1 μM; positive control) for 24 hours or chloroquine (30 μM; negative control) for 6 hours in serum-free DMEM. After incubation, autophagy was measured using a fluorescence-based Autophagy Assay Kit (abcam) according to the manufacturer’s protocol. Fluorescence images and fluorescence measurement were done with a Spark Cyto multimode reader (Tecan). Hoechst staining was used for signal normalization.

### 2.13. Spheres

Cells were seeded in a Nunclon ultra low attachment 96-well microplate (ThermoFisher Scientific) using a seeding number of 2,000 cells per well (HuCCT-1, OCUG-1, TFK-1), centrifuged for 10 minutes and 350 g and let grown for 72 hours. Cells were then incubated with ouabain (1 μM, 0.1μM or 0.01 μM) and incubated for 8 days. Spheroids were visualized on a CKX53 microscope using a XM10 camera (Olympus). Quantification of cell viability was done using a CellTiter-Glo® 3D Cell Viability Assay (Promega) according to the manufacturer’s instructions. Luminescence was measured on a Spark Cyto multimode reader (Tecan).

### 2.14. Statistics

All data points represent mean values of at least three biological replicates (n ≥ 3 independent experiments; each biological replicate contained—if applicable—an appropriate number of technical replicates) ± standard error of mean (SEM). Correlation analysis was done by calculation of the Pearson’s correlation coefficient. The paired Student’s t-test was applied for calculation of significances between control and treated samples. All calculations were performed using OriginPro 2021 (OriginLab, Northampton, MA, USA) and SPSS v24 (IBM, Armonk, New York, New York, USA). Statistical results were considered significant (*) or highly significant (**) at p < 0.05 and p < 0.01, respectively.

## 3 Results

### 3.1. Ouabain reduces cell viability of BTC cells

**Fig 1:**
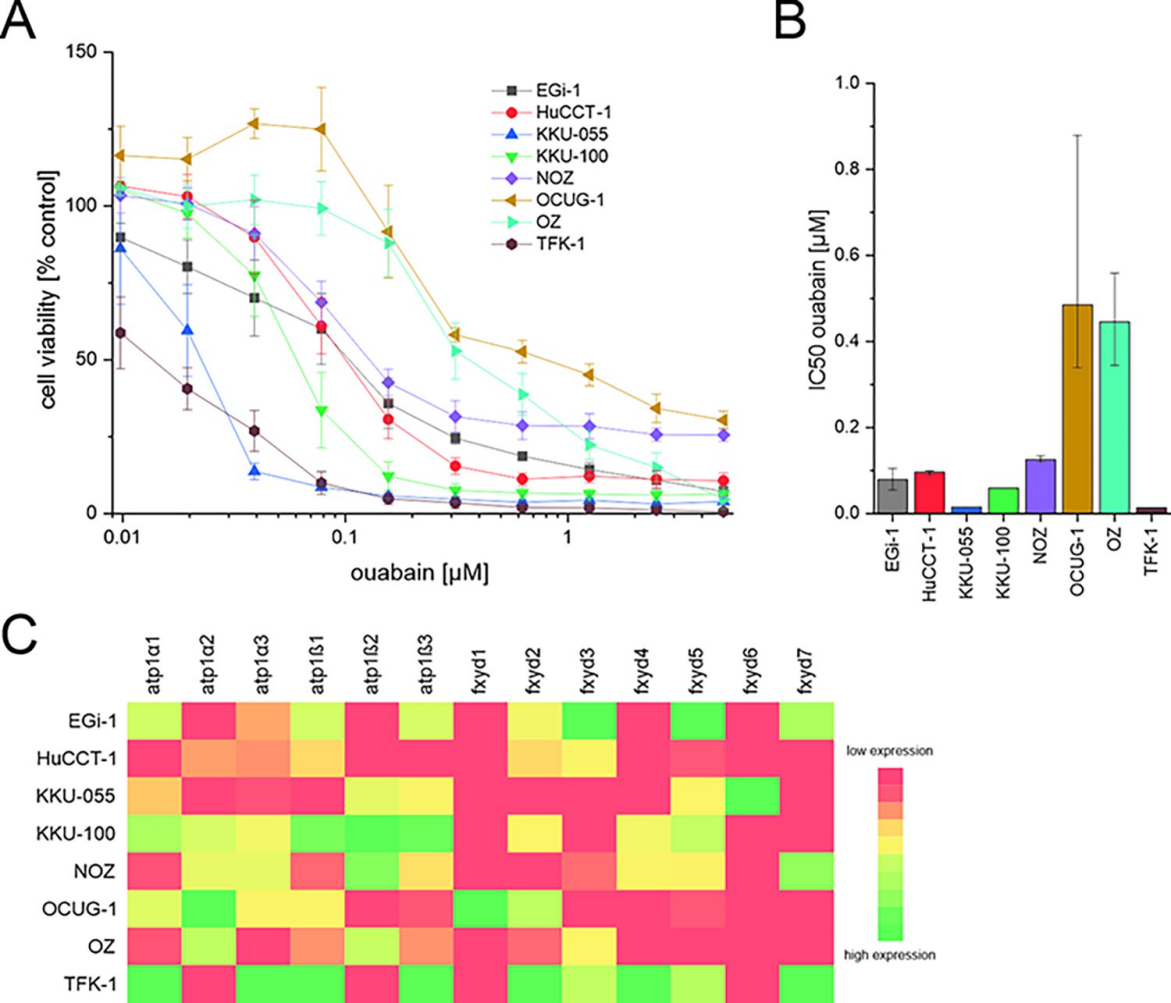
(A) BTC cells were incubated with ouabain for 72 hours. Shown are viability data related to untreated control cells as mean values ± SEM of at least n = 4 biological replicates. (B) IC_50_-values of ouabain were calculated using four-parameter logistic regression. Error bars represent the 95% confidence interval. (C) Heatmap of the expression analysis of the mRNA levels of the Na^+^/K^+^-ATPase subunits α_1–3_, β_1–3_ and fxyd_1-7_ in BTC cell lines based on the calculated ΔCt-values. Red boxes indicate low expression and green boxes indicate high expression of the respective gene normalized for each gene. For a detailed illustration of the Na^+^/K^+^-ATPase subunit expression analysis see S4 Fig. Abbreviations: BTC = biliary tract cancer.

In a first step, we investigated the cytotoxic effect of different concentrations of ouabain in BTC cells. As shown in Fig 1A, ouabain reduces the viability of BTC cells in a cell line- and concentration-dependent manner at (low) nM levels. This effect was significant for most cell lines and ouabain concentrations (see S2 Fig). Calculation of the respective IC_50_-values revealed that KKU-055 (IC_50_-value: 15 nM) and TFK-1 (14 nM) cells were most sensitive for ouabain treatment, whereas OCUG-1 (485 nM) and OZ (446 nM) were least sensitive (Fig 1B). The remaining cell lines displayed IC_50_-values between 60 nM and 126 nM. We also observed high sensitivity of MMNK-1 cells towards ouabain (S3A Fig).

### 3.2. NKA subunits are expressed in BTC cells

To evaluate a potential correlation between the sensitivity of BTC cells towards ouabain and the cell line-specific expression profile of the NKA subunits, we measured baseline mRNA levels of the four α subunits (*α*_*1*–4_), three β subunits (*β*_*1–3*_) and seven fxyd (*fxyd*_*1-7*_) in the complete set of BTC cell lines. As shown in Fig 1C and S3B and S4A Figs, NKA *α* subunits show a cell line-dependent expression profile. Of note, the α_4_ subunit was not expressed in BTC cell lines. In general, the *α*_*1*_ subunit displayed the highest expression levels of all α subunits with low expression levels in the non-tumor cholangiocytes cell line MMNK-1 (S3B Fig). Interestingly, based on data available from the GEPIA database (http://gepia.cancer-pku.cn/, accessed on 2023-03-22), of all *α* subunits, only the *α*_1_ subunit is significantly overexpressed in cholangiocarcinoma tissue compared to normal tissue on mRNA level (S3C Fig, data shown only for the *α*_1_ subunit) [39]. In contrast, the *α*_*2*_ subunit displayed the lowest expression levels of all measured *α* subunits (S4A Fig). Similarly, NKA β subunits showed a heterogeneous expression profile in BTC cell lines (Fig 1C, S4B Fig). Interestingly, the expression of the *β*_*2*_ subunit was only detectable in four of the eight tested cell lines. Of the seven fxyd isoforms, only *fxyd*_*5*_ was expressed in all cell lines (S4C Fig). The remaining fxyd isoforms were only detectable in some (*fxyd*_*2*_,_*3*_,_*4*,*7*_) or one (*fxyd*_*1*,*6*_) of the BTC cell lines. Correlation analysis revealed a highly significant positive correlation between *α*_*1*_ and *β*_*1*_ as well as *β*_*3*_ subunit expression (S5 Fig). Moreover, we found a significant positive correlation between *α*_*3*_ and *β*_*1*_, *β*_*3*_ and *fxyd*_*2*_ expression levels, respectively. In addition, we identified significant positive correlations between *β*_*1*_ and *β*_*3*_ mRNA levels as well as between mRNA levels of *fxyd*_*3*_ and *fxyd*_*5*_ (S5 Fig). However, there was no correlation between IC_50_-values and NKA mRNA subunit expression (S6 Fig).

Based on the calculated IC_50_-values, we selected OCUG-1, HuCCT-1 and TFK-1 for the subsequent experiments, as these cells are representative for cell lines with low, medium and high sensitivity towards ouabain, respectively.

### 3.3. Ouabain causes apoptosis in BTC cells

**Fig 2:**
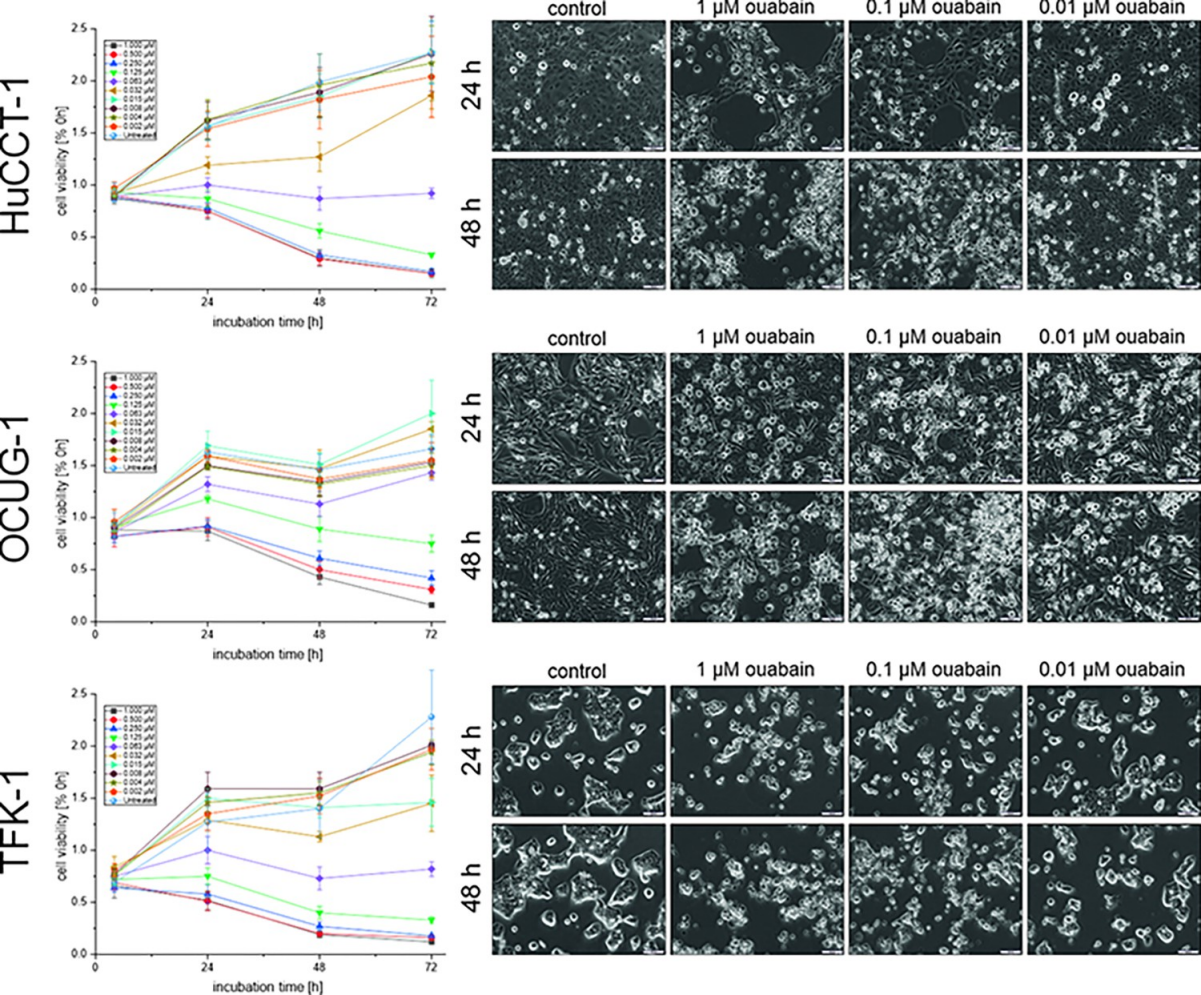
Time-resolved analysis of the effect of ouabain on the cell viability of HuCCT-1, OCUG-1 and TFK-1 cells. (left part) Viability was measured after 4, 24, 48 and 72 hours of ouabain treatment, respectively. Data represent cell viability related to the 0 hour time point. Mean values ± SEM of n = 4 biological replicates are depicted. (right part) Representative images of untreated and ouabain-treated HuCCT-1, OCUG-1 and TFK-1 cells after 24 and 48 hours, respectively; scale bars indicate 100 μm.

To get a first idea of the mode of cytotoxicity of ouabain, we performed time-resolved analysis of the effect of ouabain on cell viability. Compared to untreated cells, an effect on the viability of BTC cells was already observable after 24 hours of treatment (Fig 2, left part). In HuCCT-1 and TFK-1 cells, treatment with 0.063 μM ouabain resulted in a stop of cell growth, whereas ouabain concentrations ≥ 0.125 μM led to a relative reduction of cell viability. With respect to the higher IC_50_-value of OCUG-1, only treatment with ouabain concentrations ≥ 0.250 μM resulted in a decline of cell viability. In accordance with these data, visual assessment also revealed a cytotoxic effect of ouabain in a concentration-dependent manner beginning at 24 hours of incubation (Fig 2, right part). A statistical analysis of the results is shown in S7 Fig. Changes in the cell morphology following ouabain treatment indicate both, apoptosis and necrosis (Fig 2, right part).

**Fig 3:**
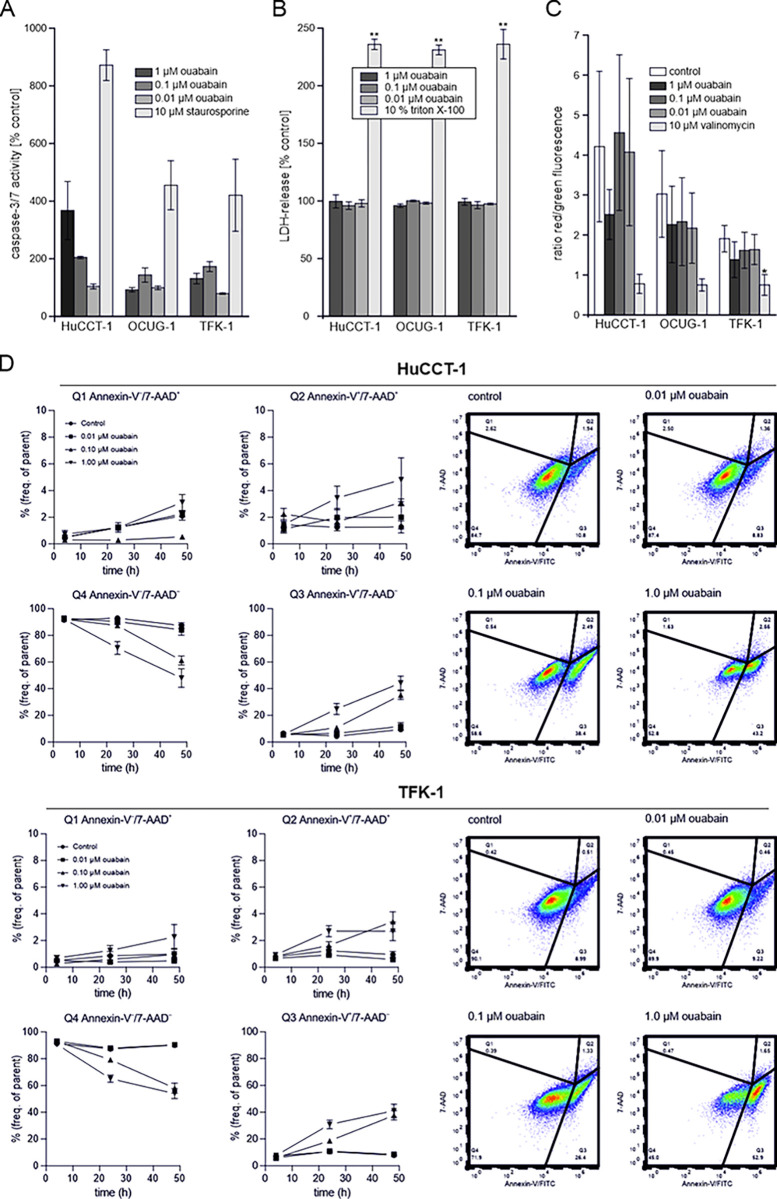
(A) Caspase 3/7 activity as indicator for apoptosis induction was measured following treatment with ouabain for 24 hours. Staurosporine was used as a positive control. Data represent mean values ± SEM of n = 3 biological replicates related to untreated controls. (B) LDH-release was measured as an indicator for necrosis following ouabain treatment for 24 hours. 10% Triton X-100 was used as a positive control. Data represent mean values ± SEM of n = 3 biological replicates related to untreated controls. (C) Changes in the mitochondrial membrane potential after treatment with ouabain were evaluated using the JC-1 dye and are presented as ratio of red to green fluorescence. A decline in the ration indicates mitochondrial membrane depolarization. Valinomycin was used as a positive control. Data represent mean values ± SEM of n = 3 biological replicates. (D) FACS-based Annexin-V/7-AAD staining was used to evaluate the time-dependent effect of ouabain on apoptosis and/or necrosis induction in HuCCT-1 and TFK-1 cells. (left part) Shown are data representing mean values ± SEM of n = 3 biological replicates. (right part) Representative plots of cell populations following ouabain treatment. Annexin-V^−^/7-AAD^−^cells (Q4) were considered as non-apoptotic, Annexin-V^+^/7-AAD^−^cells (Q3) as early apoptotic, Annexin-V^+^/7-AAD^+^ cells (Q2) as late apoptotic, and Annexin-V^–^/7-AAD^+^ cells (Q1) as post-late apoptotic/necrotic. Due to their size, OCUG-1 cells were not suitable for FACS analysis.

To further investigate the mode of action of ouabain in BTC cells, we analyzed apoptosis and necrosis induction following ouabain treatment. We found that in HuCCT-1 and TFK-1 cells, 1 μM and 0.1 μM (but not 0.01 μM) ouabain increased caspase 3/7 activities (Fig 3A), whereas treatment with the same ouabain concentrations did not lead to an increase of LDH levels (Fig 3B).

Surprisingly, we were not able to measure induction of apoptosis and/or secondary necrosis in a non-endpoint experimental setup using a RealTime-Glo^TM^ Annexin V Apoptosis and Necrosis Assay (S8 Fig). Since loss of mitochondrial membrane potential is involved in apoptosis [40], we also evaluated changes in the mitochondrial membrane potential of HuCCT-1, OCUG-1 and TFK-1 cells following treatment with 1 μM, 0.1 μM and 0.01 μM ouabain. Valinomycin was used as a positive control. As shown in Fig 3C, neither of the used ouabain concentrations caused a significant mitochondrial membrane depolarization. We next performed FACS-based measurement of Annexin-V/7-AAD staining after 4, 24 and 48 hours of treatment with 1 μM, 0.1 μM and 0.01 μM to evaluate the time dependency of the cytotoxic effect of ouabain. As illustrated in Fig 3D, 1 μM and 0.1 μM ouabain caused an increase of early and late apoptotic cells along with a reduction in non-apoptotic cells, whereas treatment with 0.01 μM ouabain did not lead to apoptotic events. Due to their size, OCUG-1 cells were not suitable for FACS analysis. Based on the findings of Trenti et al. [41], we also tested whether ouabain causes autophagy. However, following ouabain treatment, no increase in autophagy was observable (S9 Fig; only data of cells treated with 0.1 and 0.01 μM ouabain are shown, since cells treated with 1 μM ouabain were not suitable for analysis).

### 3.4. Ouabain at low nM concentrations does not cause depolarization of BTC cells and does not change intracellular sodium levels

**Fig 4:**
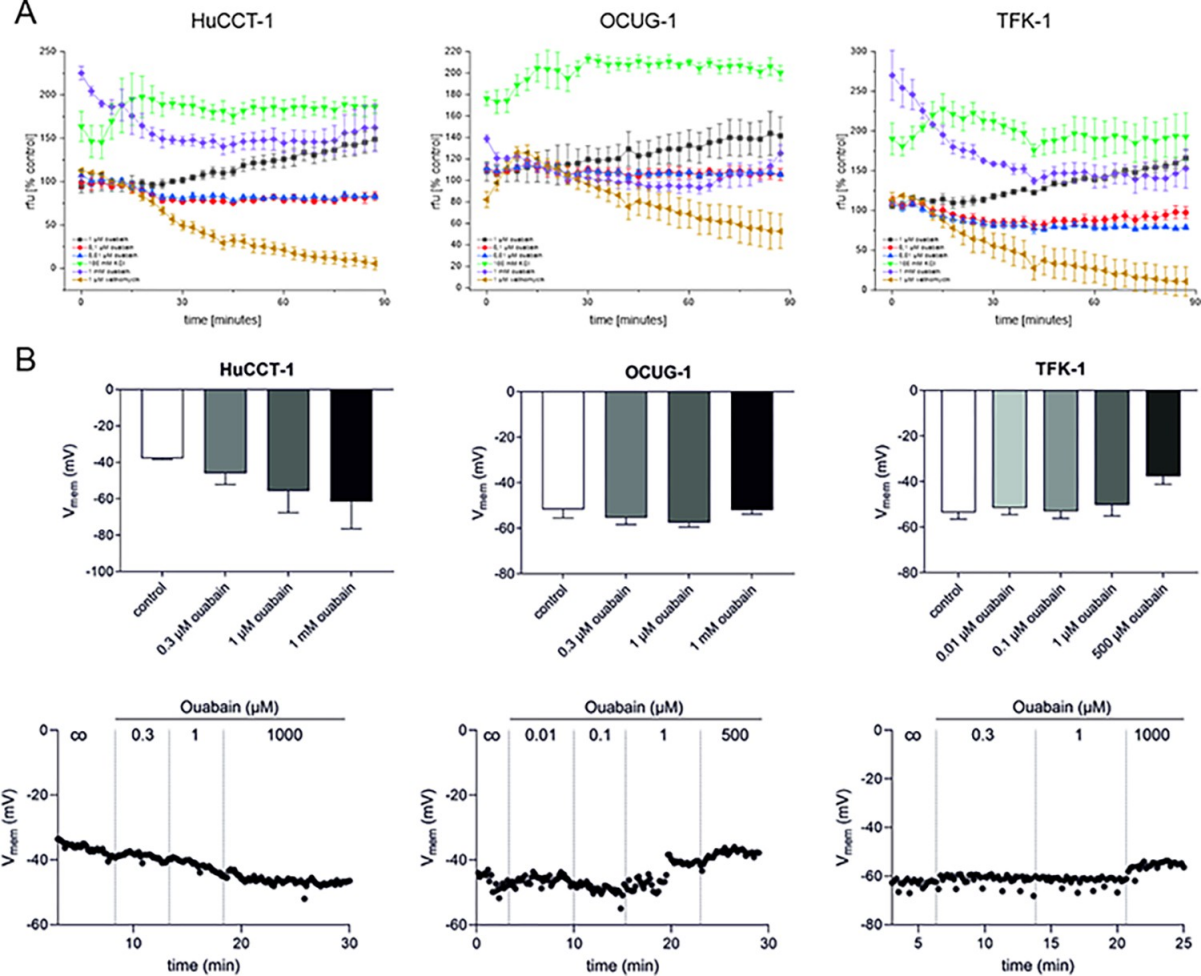
(A) The effect of different ouabain concentrations on the cell membrane potential was evaluated over a time period of 90 minutes. 1 mM ouabain and 100 mM K^+^ were used as controls for membrane depolarization, 1 μM valinomycin was used as a control for membrane hyperpolarization. An increase of fluorescence (rfu) indicates cell membrane potential depolarization, whereas a decrease indicates a hyperpolarization of the cell membrane potential. Data represent mean values ± SEM of at least n = 3 biological replicates related to untreated control cells. (B) Analysis of cell membrane potential using patch clamp (upper part). Data are represented as mean values ± SEM of three biological replicates. High ouabain concentrations (1 mM, 500 μM) were used as controls to induce membrane potential hyperpolarization. (lower part) Representative time-resolved plots of the effect of different ouabain concentrations on the membrane potential. Abbreviations: rfu = relative fluorescence units.

It was described that ouabain at sub-saturating concentrations (nM-range) causes cytotoxicity in cancer cells without disturbance of NKA activity [12, 16, 24]. We therefore measured the plasma membrane potential as well as intracellular sodium levels of HuCCT-1, OCUG-1 and TFK-1 cells following ouabain treatment as surrogate makers for NKA activity. Plasma membrane potential was measured using a Cellular Membrane Potential Kit and included valinomycin and 100 mM KCl as positive controls. Changes of the cell membrane potential were measured over a time-period of 90 minutes and are represented as relative fluorescence units (rfu), where an increase of the fluorescence signal indicates depolarization and a decrease of the fluorescence signal indicates hyperpolarization. As shown in Fig 4A, treatment of BTC cells with 0.1 and 0.01 μM ouabain did not lead to depolarization of the cell membrane potential. In contrast, incubation with 1 μM ouabain depolarizes the cell membrane potential over time as indicated by a constant increase of the fluorescence signal. Interestingly, in HuCCT-1 and TFK-1 cells, which display moderate and high ouabain sensitivity respectively, treatment with 1 mM ouabain resulted in immediate depolarization of the cell membrane potential, whereas no such effect was observable in the less ouabain-sensitive OCUG-1 cells.

Likewise, measurement of the membrane potential using the patch clamp method also shows that ouabain at low concentrations does not lead to depolarization of the cell membrane potential (Fig 4B).

**Fig 5:**
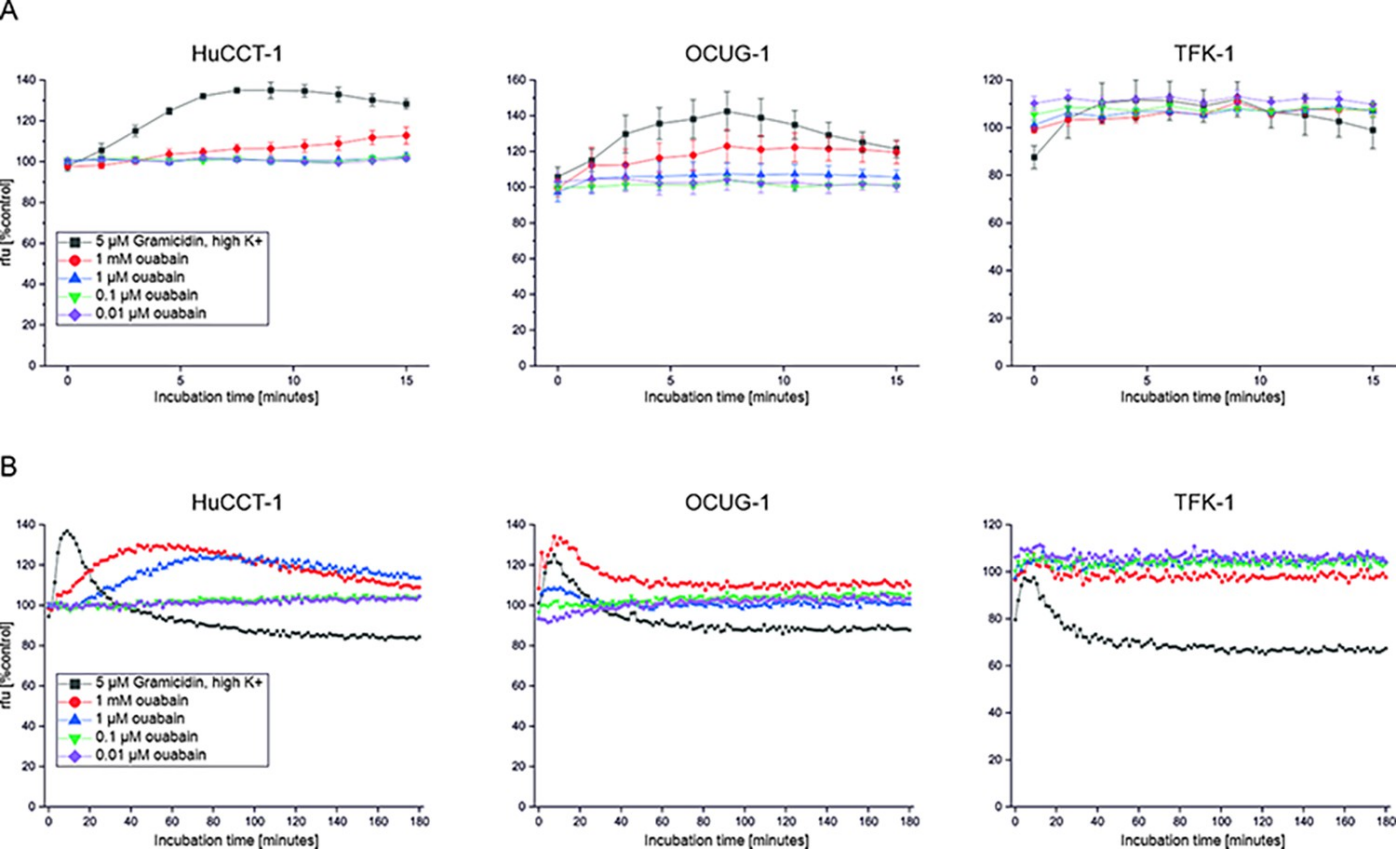
Intracellular sodium levels were measured following treatment with different concentrations of ouabain after 15 minutes (A) and 180 minutes (B), respectively. (A) Data represent fluorescence units relative to untreated control cells of mean values ± SEM of n = 3 biological replicates. (B) Representative data from one biological replicate. An increase in fluorescence (rfu) indicates increased intracellular sodium levels. Abbreviations: rfu = relative fluorescence units.

In accordance with these data, low concentrations of ouabain (0.1 μM and 0.01 μM) did not cause substantial changes to intracellular sodium levels in the tested cell lines, whereas 1 μM ouabain caused an increase of intracellular sodium levels in HuCCT-1 cells (Fig 5A and 5B). Of note, these effects were observable immediately after addition of the substances (Fig 5A) as well as after long-term measurements (Fig 5B). 5 μM Gramicidin in a high extracellular K^+^ solution and 1 mM ouabain served as positive controls.

These data indicate that the observed cytotoxic effects of 1 μM ouabain may be in a cell line-dependent manner at least partially caused by cell membrane depolarization and sodium influx, whereas the observed cytotoxic effects specifically for 0.1 μM ouabain appear to be independent of changes to the plasma membrane potential as well as of changes to intracellular sodium levels.

### 3.5. Ouabain impairs growth of BTC spheroids

**Fig 6:**
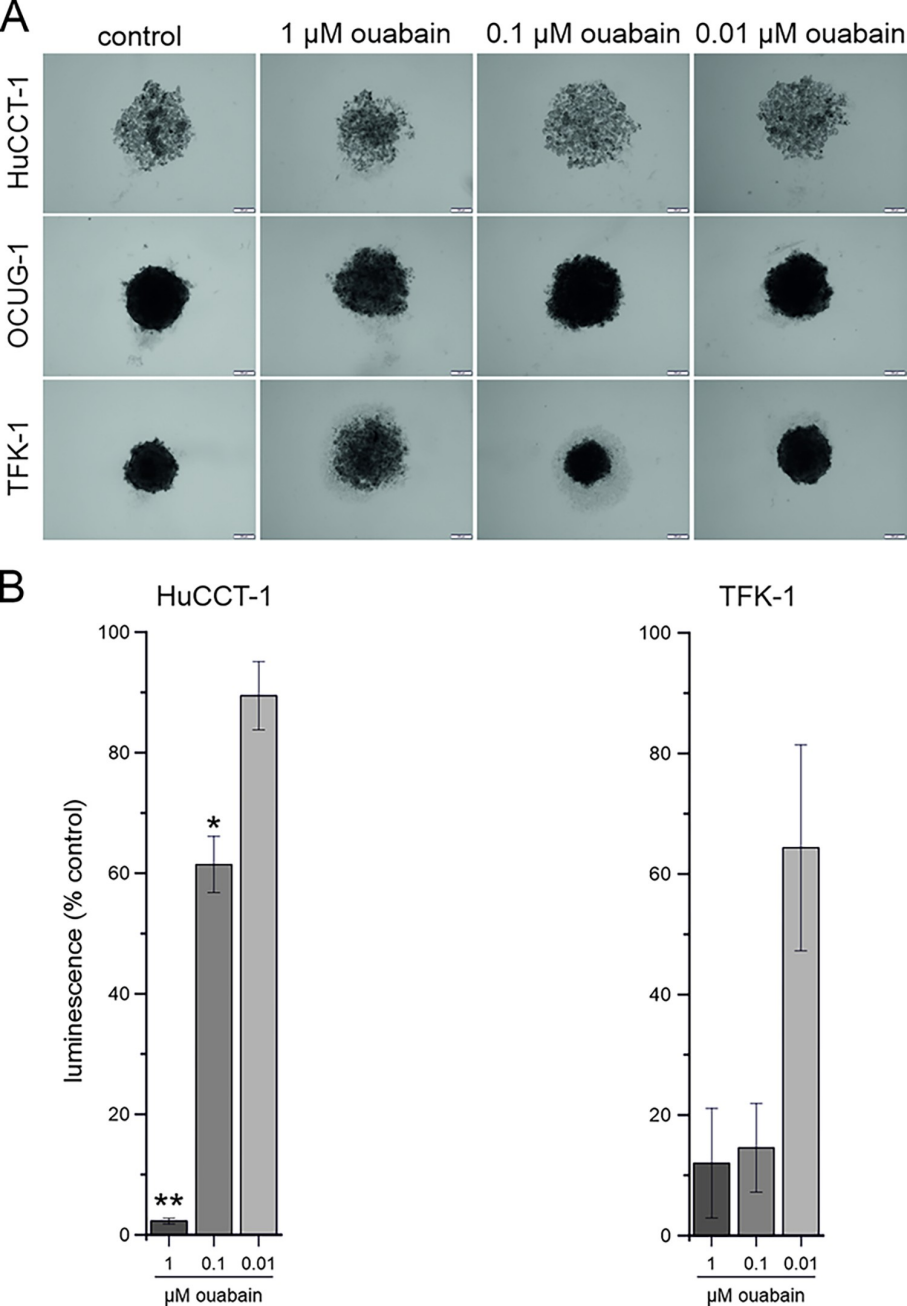
(A) Representative images of spheroids generated from HuCCT-1, OCUG-1 and TFK-1 cells, respectively, treated with different ouabain concentrations compared to untreated controls. Scale bar (yellow) indicates 200 μm for 40 × magnification. (B) Measurement of ATP levels in 3D spheroids of HuCCT-1, OCUG-1 and TFK-1 cells. Data represent luminescence units relative to untreated control cells of mean values ± SEM of n = 3 biological replicates.

Lastly, we investigated the effect of ouabain on the growth of BTC 3D-spheroids to better mimic pathophysiological conditions. In general, we found that OCUG-1 and TFK-1 cells were able to form optically compact and dense spheres, whereas HuCCT-1-derived spheres were less dense and more loosely structured. As shown in Fig 6A, treatment of spheroids of HuCCT-1, OCUG-1 and TFK-1 cells with ouabain led to morphological changes such as fringing and decompaction of the spheres in a concentration-dependent manner. Additionally, we quantified the effect of the different ouabain concentrations on the viability of the cells in of the 3D spheroids. Treatment with 1 μM and 0.1 μM led to a (significant) reduction of ATP levels in spheroids of all tested cell lines (Fig 6B).

## 4 Discussion

In the present work, we provide first data regarding the potential of ouabain as an anti-BTC substance. We found that in a cell line-dependent manner, ouabain reduces the viability of BTC cells *in vitro* with IC_50_-values in the (low) nM-range, where the most sensitive cell line displayed an IC_50_-value of 14 nM and the least sensitive cell line of 485 nM. Compared to findings in other cancer entities, these data suggest that BTC cells might display a general high sensitivity towards ouabain treatment, although the effects of ouabain on the cell viability are strongly cell line-dependent. Chang et al. found that ouabain reduced the viability of prostate cancer cells in the μM-range [42]. In glioma cells, IC_50_-values of ouabain of about 500 nM are described [29, 43]. In breast cancer cells, in a study by Kometiani et al. cytotoxic effects of ouabain were observed only at concentrations of 250 nM and above [24]. However, there are also reports of tumor cell lines that display similar sensitivity towards ouabain treatment compared to our *in vitro* model. For example, Khajah et al. calculated IC_50_-values ranging from about 5 to 150 nM for five different breast cancer cell lines [27]. Likewise, A549 cells displayed an IC_50_-value at a low nM concentration of ouabain [26]. Regarding the mode of cytotoxicity, we found that ouabain mainly causes apoptosis in BTC cells, which is in accordance with other studies that described induction of apoptosis following treatment with ouabain [27, 42]. However, our data regarding the mode of cytotoxicity of ouabain in BTC cells were not fully conclusive. This might be explainable by phenomenons termed ‘hybrid cell death’ or ‘oncosis’, which are described as forms of cell death that are composed of characteristics from apoptotic and necrotic cell death [25, 44]. Interestingly, in glioblastoma cells, induction of hybrid cell death has already been demonstrated following ouabain treatment [25].

As mentioned earlier, sub-saturating concentrations of ouabain might exert their effects via activation of intracellular signaling pathways independent of inhibition of the pumping function of the NKA. In fact, potential effects of ouabain on the NKA pumping function can be measured as soon as 10 minutes after addition in breast cancer cells [24]. In our experiments, we observed significant cytotoxic effects of ouabain at nM and low μM concentrations and found for 0.1 and 0.01 μM that these effects were mostly independent of changes of the cellular membrane potential and changes in intracellular sodium levels, suggesting that these ouabain concentrations might not interfere with NKA activity, but rather induce activation of the NKA as a signal transducer. In this regard, Shen et al. demonstrated activation of the AMPK and Src pathways already after 15 minutes of incubation with 25 nM ouabain in breast and lung cancer cells [26]. Moreover, Kometiani et al. also demonstrated no increase in intracellular Na^+^ levels following incubation of breast cancer with 0.1 μM ouabain for 30 minutes [24]. However, direct measurement of NKA pumping activity as well as detailed investigation of putatively involved signaling pathways in ouabain-caused cytotoxicity in BTC cells are required to fully clarify the potential of ouabain as an anti-BTC substance.

Although ouabain had a potent general cytotoxic effect in our *in vitro* model, we observed strong cell-line dependent differences regarding the sensitivities of the used BTC cells. However, we found no correlation of the IC_50_-values and the expression levels of the different NKA subunits, although we observed a strongly cell line-dependent expression pattern of the different *α*, *β* and *fxyd* subunits. Interestingly, the *atp1α*_*1*_ subunit was expressed in all cell lines at relative high levels. This might be due to the fact, that the *atp1α*_*1*_ subunit is generally expressed in a variety of tissues, whereas the other α-subunits are expressed more in a tissue-specific manner [12, 14]. Likewise, the *β*_*1*_ and *β*_*3*_ subunits were expressed in all tested BTC cell lines, which might also be explainable by their general expression in most human tissues [12]. Although we found no correlation between NKA subunit expression and ouabain sensitivity in our *in vitro* model, there is evidence that differences in the expression levels of specific NKA subunits might have implications regarding the viability of tumor cells. Nakamura et al. not only demonstrated that *atp1α1* was strongly expressed in gastric cancer cells, but also that knockdown of *atp1α*_*1*_ resulted in induction of apoptosis [45]. Moreover, high protein levels of *atp1α*_*1*_ correlated with poor prognosis in gastric cancer [45]. Likewise, publically available data regarding the expression of atp1*α*_*1*_ in cholangiocarcinoma samples suggest elevated levels compared to healthy tissue—in line with the observation that MMNK-1 cells displayed low mRNA levels (see S3B Fig). Differences in NKA subunit expression might also be involved in chemoresistance. Chen et al. analyzed NKA subunit expression in chemo-sensitive and chemo-resistant glioma cells and found high levels of *atp1α2* and *atp1α3* in the resistant cells [25]. Moreover, they found that knockdown of the *atp1α3* subunit sensitized cells towards chemotherapeutic treatment [25]. For BTC, future studies should therefore analyze protein levels of the different NKA α subunits in patient samples and investigate a potential correlation between expression levels and the sensitivity towards standard chemotherapeutics.

Regarding the *fxyd* subunits, mRNA expression levels showed a strong cell line-dependency, meaning that specific *fxyd* subunits were only expressed in some or one BTC cell line(s). However, we found that *fxyd5* was expressed in all tested BTC cell lines. *Fxyd5* is also known as *dysadherin* and although *fxyd5* was shown to directly interact with the *α* subunit of the NKA, it has also been demonstrated that it is involved in cancer progression. This observation is underlined by the fact that *fxyd5* is expressed in tumor cells of various origin, while it was only detectable in a limited number of non-tumor cell lines [46]. Moreover, there is evidence that *fxyd5* plays a role cell adhesion. Forced expression of *fxyd5* in liver cancer cells resulted in reduced cell adhesion and diminished *E-cadherin* levels [46]. In addition, high levels of *fxyd5* correlated with unfavorable clinical parameters in different tumor types and studies. Interestingly, in these studies, an inverse correlation between *fxyd5* and *E-cadherin* expression was not always observed, suggesting additional tumorigenic effect of *fxyd5* independent of the *fxyd5* / *E-cadherin* connection [47].

In our *in vitro* model, treatment with ouabain had a significant effect on viability of BTC cells grown as spheroids. In this regard, Guo et al. also demonstrated an effect of ouabain on spheroid formation and growth of osteosarcoma cells, although the experimental setup and readouts were different [48]. Interestingly, they measured a significant reduction of the size of the spheres, whereas in the BTC cells, we primarily observed structural changes such as fringing and decompaction as well as significantly reduced ATP levels.

In summary, we demonstrate that concentrations of ouabain at (low) nM concentrations have a potent cytotoxic effect on BTC cells independent of depolarization of the cell membrane potential and changes to intracellular sodium levels. Together with our observation that ouabain impairs spheroid growth and viability of spheroidal cells, we suggest that ouabain as an anti-BTC substance should be further investigated, specifically the involvement of specific signaling pathways as well as the effects of ouabain on functional tumor characteristics in BTC cells.

## Supporting information

S1 Fig List of the used primers for Na^+^/K^+^-ATPase (NKA) subunits and the reference gene beta-actin (*actb*). (PDF)

S2 Fig Statistics for Fig 1A. Significant differences in cell viability between treated and untreated samples were calculated by paired t-tests. * (light green) indicate significant (p < 0.05) and ** (dark green) indicate highly significant (p <0.01) results, respectively.
(PDF)

S3 Fig (A) MMNK-1 cells were incubated with ouabain for 72 hours. Shown are viability data related to untreated control cells as mean values ± SEM of n = 3 biological replicates. (B) Analysis of mRNA levels of the Na^+^/K^+^-ATPase (NKA) α_1_ subunit in BTC cells and non-tumor MMNK-1 cholangiocytes (highlighted in red). Data are presented as mean values of at least n = 3 biological replicates ± SEM related to mRNA levels of beta-actin. (C) Expression analysis of the (NKA) α_1_ subunit in cholangiocarcinoma patient samples compared to normal tissue based on the GEPIA database (39). Abbreviations: BTC = biliary tract cancer; CHOL = cholangiocarcinoma; N = normal tissue; T = tumor tissue.
(TIF)

S4 Fig Analysis of mRNA levels of Na+/K+-ATPase (NKA) α subunits (A), β subunits, and fxyd subunits (C) in biliary tract cancer cell lines. Data are presented as mean values of n = 3 biological replicates ± SEM related to mRNA levels of beta-actin.
(TIF)

S5 Fig Correlation analysis (Pearson) between mRNA levels of Na^+^/K^+^-ATPase (NKA) subunits. Correlation analysis for fxyd1 and fxyd6 was not possible due to missing data points. * (light green) indicate significant (p < 0.05) and ** (dark green) indicate highly significant (p <0.01) results, respectively.
(PDF)

S6 Fig Correlation analysis (Pearson) between mRNA levels of Na^+^/K^+^-ATPase (NKA) subunits and IC_50_. Correlation analysis.
(PDF)

S7 Fig Statistics for Fig 2. Significant differences in cell viability between treated and untreated samples were calculated by paired t-tests. * (light green) indicate significant (p < 0.05) and ** (dark green) indicate highly significant (p <0.01) results, respectively.
(PDF)

S8 Fig Non-endpoint measurement of phosphatidylserine translocation and membrane integrity under cell incubator conditions (5% CO2, 37°C, humidity) to evaluate apoptosis and secondary necrosis in HuCCT-1, OCUG-1, and TFK1- cells following treatment with ouabain. Data are presented as mean values of n = 4 biological replicates ± SEM. According to the manufacturer’s instructions, a strong initial increase in the luminescence signal (indicating phosphatidylserine presence in the outer leaflet of the plasma membrane) followed by a time-delayed increase of the fluorescence signal (representing loss of membrane integrity) would indicate apoptosis followed by secondary necrosis.
(TIF)

S9 Fig Evaluation of induction of autophagy in HuCCT-1, OCUG-1 and TFK-1 cells following treatment with ouabain. Data represent normalized fluorescence units (green fluorescence representing autophagic events normalized to Hoechst DNA stain) of mean values of n = 3 biological replicates ± SEM.
(TIF)
