# Supplementary material for: Ouabain at nanomolar concentrations is cytotoxic for biliary tract cancer cells
Source: PLoS One. 2023 Jun 30;18(6):e0287769. doi: 10.1371/journal.pone.0287769 (PMC10312999; doi:10.1371/journal.pone.0287769)
Supplement: S1 Fig — (PDF) [file pone.0287769.s001.pdf]

| Gene Name | Forward Primer (5'-3') | Reverse Primer (5'-3') |
|---|---|---|
| FXYD1 | ACATCTTGGTTTTCTGTGTG | ACTGGTAGTCGTAAGTGAAC |
| FXYD2 | TATGACTATGAGACCGTTCG | ATCTTCATTGATTTGCCTGC |
| FXYD3 | CTTCTGCTGATCCTGAAATTG | CCTCACTTCTTTTCCTTAGATG |
| FXYD4 | CTACTATGACTGGAAAAACCTG | ATTTGCATTTGCCACTCAG |
| FXYD5 | CTCTAGTGACAGATCCAGAG | GTGTTCATCATAGAAGAAGGG |
| FXYD6 | TATGATTACCAGACCCTGAG | CTGCGACTTAGGATAAGGAG |
| FXYD7 | CCTGACCCATTTTACTATGAC | GCTCAGACTTACAGGATTTG |
| ATP1A1 | ATACGACAGAGAATCAGAGTG | TAGGTAGGTTTTCCTGGTTAG |
| ATP1A2 | AATCTATATCTGGGTGTGGTG | GTACCATGTTCTTGAAGGAATC |
| ATP1A3 | ATGACCTGGAAGACAGTTAC | CAGGATCTTGTTCTTCATGC |
| ATP1A4 | TCCCTACAGTATTCTCATCTTC | CTAGTGTCTCAGATGTTGTG |
| ATP1B1 | CCAGGATTAACACAGATTCC | GTTCAGTACATATGCCTCATAG |
| ATP1B2 | CAAGACTGAGAACCTTGATG | CTTGGATAGAGTCGTTGTAAG |
| ATP1B3 | GATTAAAGCCTGAAGGAGTG | GAGGATAAACTGCTACATTTGG |
| beta-actin | GACGACATGGAGAAAATCTG | ATGATCTGGGTCATCTTCTC |
