# Supplementary figures and images for: Ouabain at nanomolar concentrations is cytotoxic for biliary tract cancer cells
Source: PLoS One. 2023 Jun 30;18(6):e0287769. doi: 10.1371/journal.pone.0287769 (PMC10312999; doi:10.1371/journal.pone.0287769)

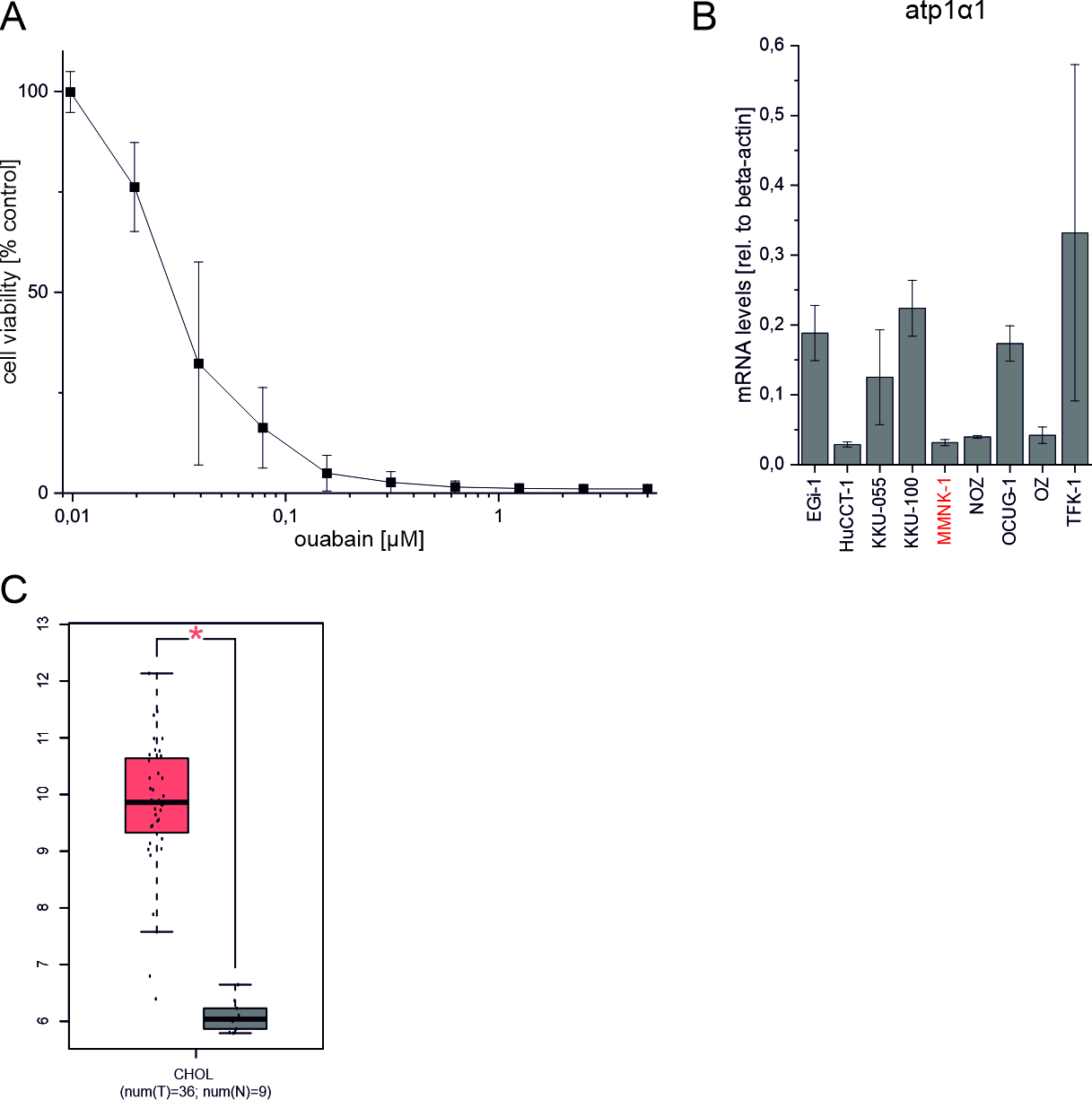

Supplement: S3 Fig — (A) MMNK-1 cells were incubated with ouabain for 72 hours. Shown are viability data related to untreated control cells as mean values ± SEM of n = 3 biological replicates. (B) Analysis of mRNA levels of the Na+/K+-ATPase (NKA) α1 subunit in BTC cells and non-tumor MMNK-1 cholangiocytes (highlighted in red). Data are presented as mean values of at least n = 3 biological replicates ± SEM related to mRNA levels of beta-actin. (C) Expression analysis of the (NKA) α1 subunit in cholangiocarcinoma patient samples compared to normal tissue based on the GEPIA database (39). Abbreviations: BTC = biliary tract cancer; CHOL = cholangiocarcinoma; N = normal tissue; T = tumor tissue. (TIF) [file pone.0287769.s003.tif]

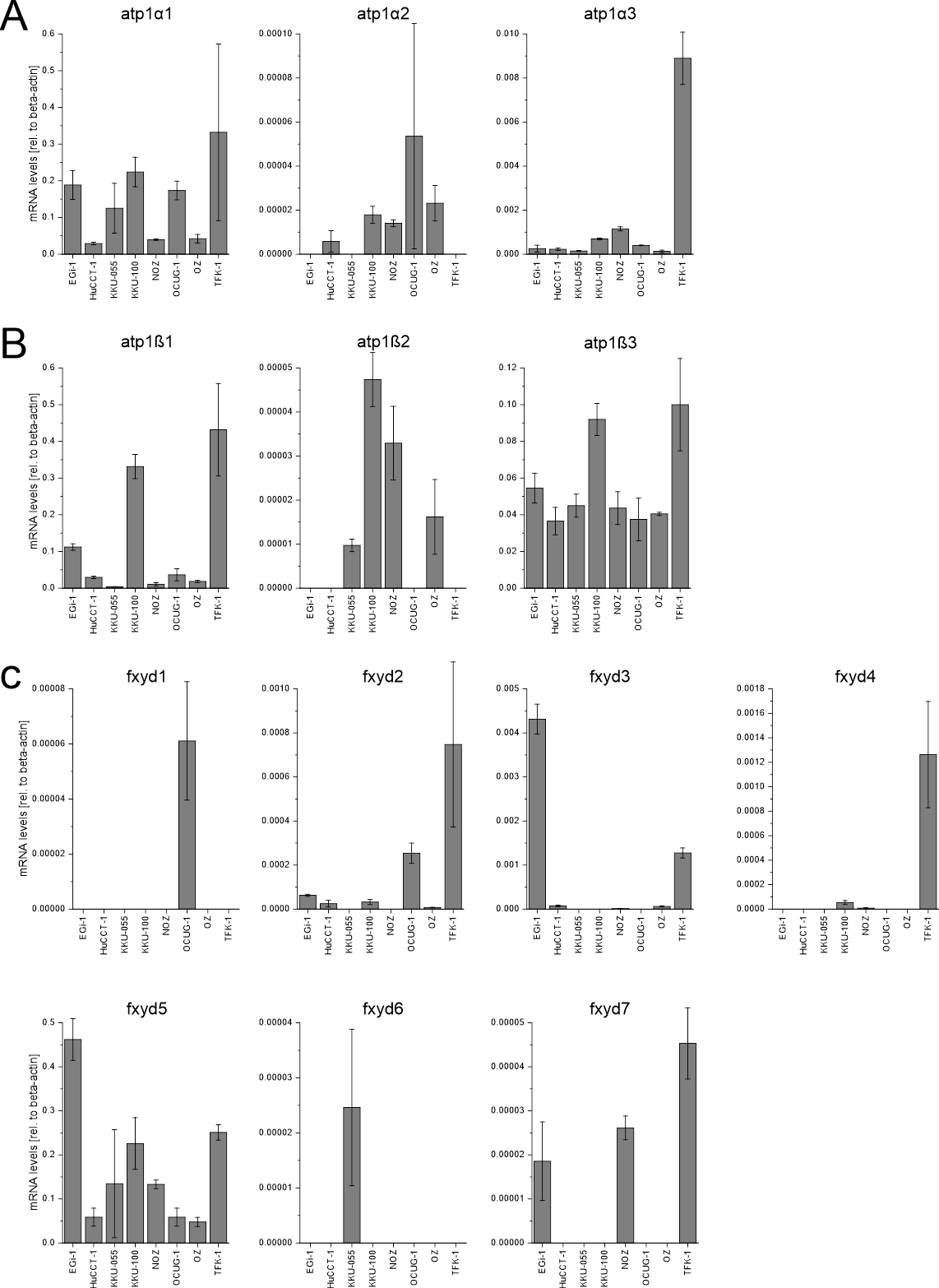

Supplement: S4 Fig — Analysis of mRNA levels of Na+/K+-ATPase (NKA) α subunits (A), β subunits, and fxyd subunits (C) in biliary tract cancer cell lines. Data are presented as mean values of n = 3 biological replicates ± SEM related to mRNA levels of beta-actin. (TIF) [file pone.0287769.s004.tif]

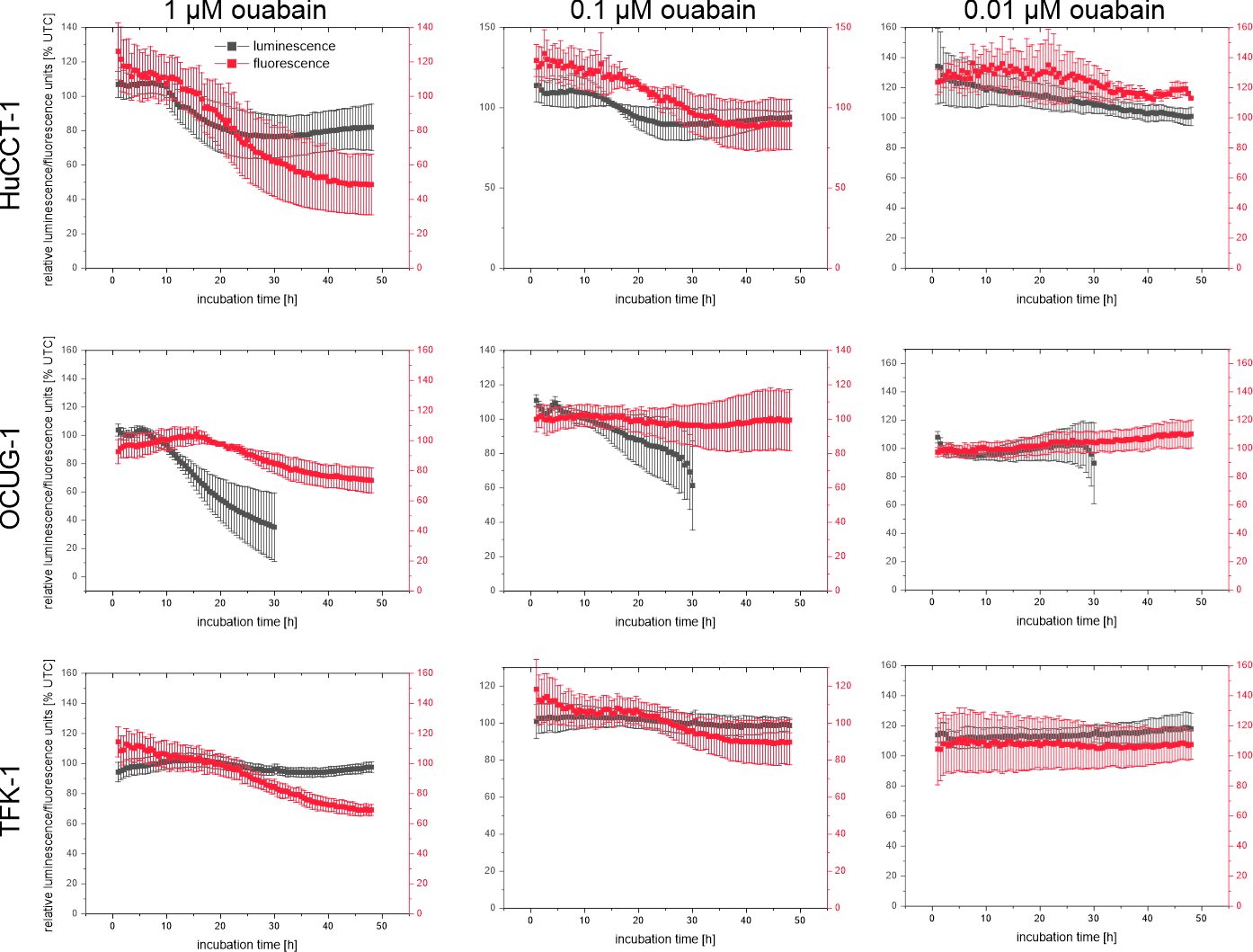

Supplement: S8 Fig — Data are presented as mean values of n = 4 biological replicates ± SEM. According to the manufacturer’s instructions, a strong initial increase in the luminescence signal (indicating phosphatidylserine presence in the outer leaflet of the plasma membrane) followed by a time-delayed increase of the fluorescence signal (representing loss of membrane integrity) would indicate apoptosis followed by secondary necrosis. (TIF) [file pone.0287769.s008.tif]

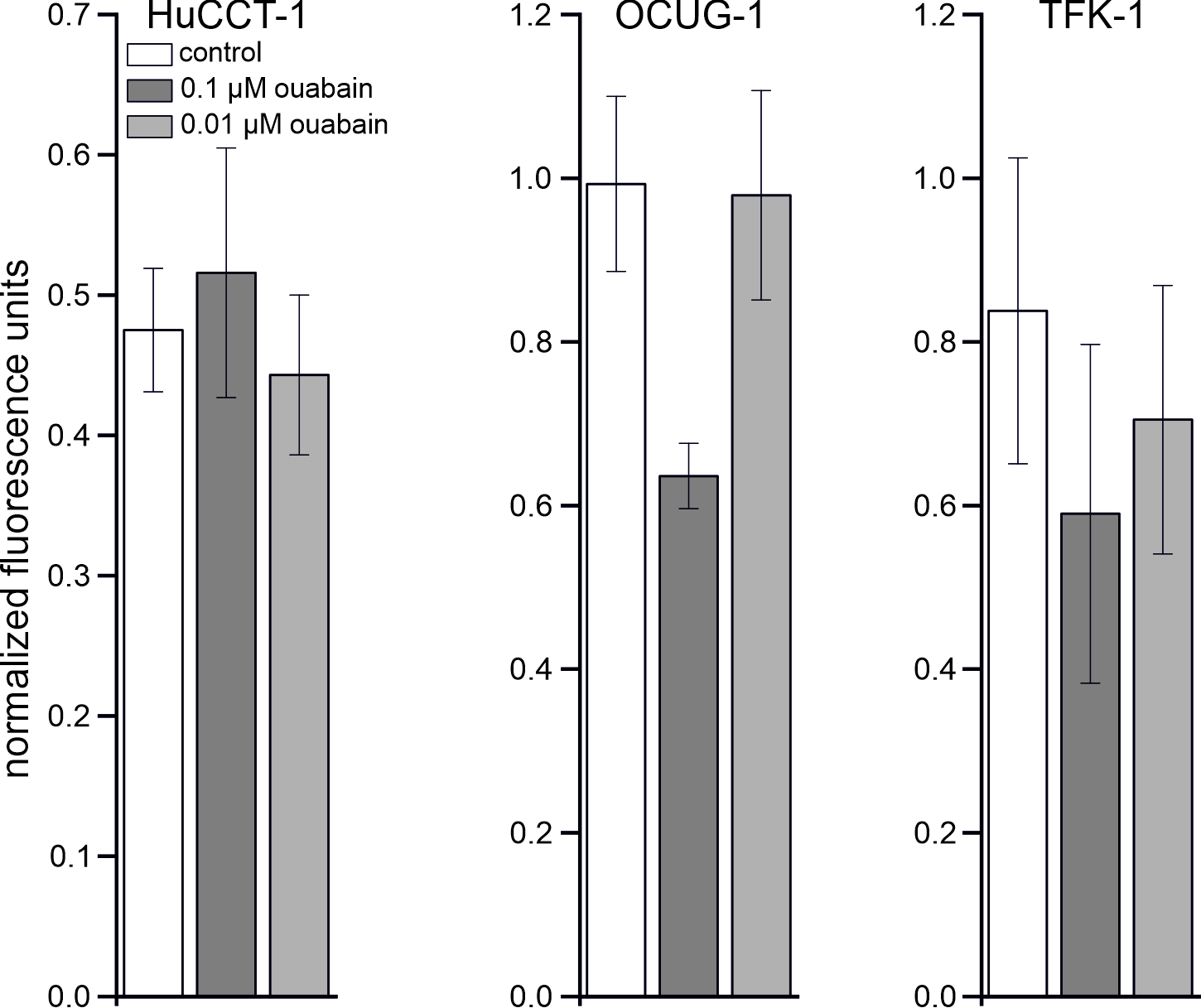

Supplement: S9 Fig — Data represent normalized fluorescence units (green fluorescence representing autophagic events normalized to Hoechst DNA stain) of mean values of n = 3 biological replicates ± SEM. (TIF) [file pone.0287769.s009.tif]
