# Supplementary material for: Ouabain at nanomolar concentrations is cytotoxic for biliary tract cancer cells
Source: PLoS One. 2023 Jun 30;18(6):e0287769. doi: 10.1371/journal.pone.0287769 (PMC10312999; doi:10.1371/journal.pone.0287769)
Supplement: S5 Fig — Correlation analysis for fxyd1 and fxyd6 was not possible due to missing data points. * (light green) indicate significant (p < 0.05) and ** (dark green) indicate highly significant (p <0.01) results, respectively. (PDF) [file pone.0287769.s005.pdf]

		atp1α2	atp1α3	atp1β1	atp1β2	atp1β3	fxyd2	fxyd3	fxyd4	fxyd5	fxyd7
"atp1α1"	Pearson Corr.	0,5086	0,7058	0,8648	0,5344	0,8414	0,7641	0,5283	0,8015	0,5665	0,6883
	p-value	0,3816	0,0504	**<0,01	0,4656	**<0,01	0,0769	0,3601	0,4081	0,1432	0,5167
"atp1α2"	Pearson Corr.		-0,1735	-0,1128	-0,6118	-0,1805	0,9140	-0,1567	1,0000	-0,2711	--
	p-value		0,7802	0,8567	0,5809	0,7715	0,0860	0,8998	--	0,6592	--
"atp1α3"	Pearson Corr.			0,7673	0,7201	0,7203	0,9512	0,0090	0,9967	0,2313	0,9833
	p-value			*0,02627	0,2799	*0,04387	**<0,01	0,9886	0,0519	0,5816	0,1165
"atp1β1"	Pearson Corr.				0,8218	0,9827	0,6630	0,2628	0,7071	0,4591	0,8723
	p-value				0,1782	**<0,01	0,1512	0,6693	0,5000	0,2525	0,3253
"atp1β2"	Pearson Corr.					0,8133	1,0000	-1,0000	1,0000	0,7531	--
	p-value					0,1867	--	--	--	0,2469	--
"atp1β3"	Pearson Corr.						0,5802	0,2751	0,6347	0,4896	0,8954
	p-value						0,2274	0,6542	0,5622	0,2182	0,2938
"fxyd2"	Pearson Corr.							0,0098	1,0000	0,1400	1,0000
	p-value							0,9902	--	0,7914	--
"fxyd3"	Pearson Corr.								1,0000	0,9662	-0,4891
	p-value								--	**<0,01	0,6746
"fxyd4"	Pearson Corr.									0,6895	1,0000
	p-value									0,5157	--
"fxyd5"	Pearson Corr.										-0,4254
	p-value										0,7203

Calculation of the Pearson correlation coefficient for fxyd1 and fxyd6 was not possible due to missing data points
