# Supplementary material for: Ouabain at nanomolar concentrations is cytotoxic for biliary tract cancer cells
Source: PLoS One. 2023 Jun 30;18(6):e0287769. doi: 10.1371/journal.pone.0287769 (PMC10312999; doi:10.1371/journal.pone.0287769)
Supplement: S6 Fig — Correlation analysis. (PDF) [file pone.0287769.s006.pdf]

		atp1α1	atp1α2	atp1α3	atp1β1	atp1β2	atp1β3	fxyd2	fxyd3	fxyd4	fxyd5	fxyd7
"IC50_ouabain"	Pearson Corr.	-0,3186	0,7955	-0,3400	-0,4368	-0,2863	-0,5119	-0,2496	-0,3822	-0,8252	-0,5320	-0,7622
	p-value	0,4418	0,1076	0,4100	0,2792	0,7137	0,1947	0,6334	0,5254	0,3822	0,1747	0,4483
