# Supplementary material for: Ouabain at nanomolar concentrations is cytotoxic for biliary tract cancer cells
Source: PLoS One. 2023 Jun 30;18(6):e0287769. doi: 10.1371/journal.pone.0287769 (PMC10312999; doi:10.1371/journal.pone.0287769)
Supplement: S7 Fig — Significant differences in cell viability between treated and untreated samples were calculated by paired t-tests. * (light green) indicate significant (p < 0.05) and ** (dark green) indicate highly significant (p <0.01) results, respectively. (PDF) [file pone.0287769.s007.pdf]

| Ouabain [µM] | | 1,000 | 0,500 | 0,250 | 0,125 | 0,063 | 0,031 | 0,016 | 0,008 | 0,004 | 0,002 |
|---|---|---|---|---|---|---|---|---|---|---|---|
| **HuCCT-1** | 4 h | | | | | | | | | | |
| | 24 h | ** | ** | ** | ** | ** | ** | | | | |
| | 48 h | ** | ** | ** | ** | * | * | | | | |
| | 72 h | * | * | * | * | | | | | | |
| **OCUG-1** | 4 h | | | | | | | | | | |
| | 24 h | ** | ** | ** | * | | | | * | | * |
| | 48 h | ** | ** | ** | ** | | | * | | | |
| | 72 h | ** | ** | ** | * | | * | * | | ** | |
| **TFK-1** | 4 h | | | | | * | | | | * | |
| | 24 h | * | | | | | | | | | |
| | 48 h | ** | ** | ** | ** | * | | | * | ** | |
| | 72 h | * | * | * | * | | | | | | |
